# Hypermobile Anterior Horn of the Lateral Meniscus: A Retrospective Case-Series Study of Presentation, Imaging, Treatment, and Outcomes

**DOI:** 10.3390/medicina60091497

**Published:** 2024-09-13

**Authors:** Chang-Hao Lin, Chen-Hao Chiang, Wei-Chen Hung, Wei-Hsing Chih

**Affiliations:** 1 Department of Orthopaedics, Ditmanson Medical Foundation Chia-Yi Christian Hospital, Chiayi 600, Taiwan; cych12763@gmail.com (C.-H.L.); chiangabaca@gmail.com (C.-H.C.); dan80215@gmail.com (W.-C.H.); 2 Department of Microbiology, Immunology and Biopharmaceuticals, College of Life Sciences, National Chia-Yi University, Chiayi 600, Taiwan

**Keywords:** arthroscopy, hypermobility, lateral meniscus

## Abstract

*Background and Objectives*: Hypermobility of the lateral meniscus is typically associated with the posterior part of this structure, with occurrences in the anterior part rarely reported. However, a hypermobile anterior horn of the lateral meniscus can manifest clinical symptoms. This study aimed to increase awareness regarding hypermobility in the anterior horn of the lateral meniscus by presenting its clinical presentations, magnetic resonance imaging (MRI) findings, arthroscopic findings, treatment approaches, postoperative protocols, and clinical outcomes. *Materials and Methods*: A retrospective case-series involving patients diagnosed as having hypermobile anterior horn of the lateral meniscus through arthroscopy. The clinical presentations, preoperative image findings, arthroscopic findings, treatments, postoperative protocols, and clinical outcomes following meniscal stabilization were all reviewed. *Results*: A total of 17 patients (17 knees) with a mean age of 45.9 ± 18.4 years were analyzed. The mean follow-up period was 18.2 ± 7.6 months (range, 6–24 months). Primary symptoms included anterior lateral knee pain, tenderness in the lateral joint lines, and a locking sensation in six of the knees. MRI revealed hypodense lesions anterior to the meniscus, fluid accumulation, degenerative changes, and anterior horn deformities. Following meniscal stabilization, the Lysholm Knee Scoring Scale score increased from 65.8 ± 12.7 before surgery to 91.1 ± 9.6 at the final follow-up (*p* < 0.001). All the analyzed knees achieved a full range of motion by the final follow-up, with no patient experiencing any complication or requiring reoperation. *Conclusions*: There is no specific sign or test that can be used to detect a hypermobile anterior horn of the lateral meniscus. A thorough arthroscopic examination is essential for diagnosing hypermobility in the anterior horn of the lateral meniscus. Arthroscopic meniscal stabilization yields favorable outcomes.

## 1. Introduction

Hypermobility in the lateral meniscus is recognized as a contributing factor of knee pain and the occurrence of locking or a restricted range of motion during knee flexion. Notably, hypermobility can occur even in the absence of detectable meniscal tears in magnetic resonance images. A hypermobile lateral meniscus is diagnosed following the identification of a translation or movement beyond the midpoint of the lateral condyle or tibia during arthroscopy [1,2,3,4,5,6,7,8].

The majority of reported hypermobile meniscus cases have occurred in the posterior horn of the lateral meniscus [1,2,3,4,5,6,7,8], with only one case report detailing hypermobility in the anterior horn of the lateral meniscus [9]. Surgical interventions for treating a hypermobile meniscus include meniscal stabilization through suturing devices, arthroscopic thermal shrinkage, and partial meniscectomy [4,6,7,8,9].

The objective of this study was to outline the characteristics of a hypermobile anterior horn of the lateral meniscus. To achieve this objective, this study explored this condition’s clinical presentation, imaging manifestations, arthroscopic findings, treatment modalities, postoperative protocols, and clinical outcomes following meniscal stabilization.

## 2. Materials and Methods

### 2.1. Patients

The study design was approved by our hospital’s institutional review board, which waived the requirement to obtain informed consent. Patients who received a diagnosis of hypermobility in the anterior part of the lateral meniscus during arthroscopy between January 1993 and February 2023 were consecutively included. Patients with discoid morphology, tear of lateral meniscus, gout, a follow-up period of <6 months, and a history of meniscus surgery on the affected knee were excluded.

### 2.2. Data Collection

After patient identification, the medical records were retrospectively reviewed to collect information regarding age, sex, history of trauma, preoperative symptoms, preoperative findings from plain film and magnetic resonance imaging (MRI), surgical procedures, and arthroscopic findings. The Lysholm Knee Scoring Scale scores were assessed at 6, 12, and 24 months after the surgery [10].

### 2.3. Surgical Procedure

Each patient underwent a knee arthroscopy conducted by an experienced surgeon. During inspection of the lateral compartment, the lateral meniscus was examined and probed. The surgeon assessed meniscal mobility during probing. Specifically, the probe was hooked anterior to the anterior horn of the lateral meniscus, and posterior pressure was applied. Hypermobility was defined as the translation of the entire anterior horn beyond the midpoint of the tibial articular surface. In all cases, hypermobility was readily discernible with mild stress and, in many cases, it was observable by manipulating the knee into a figure-of-four position. To achieve stabilization, the anterior horn of the lateral meniscus was fixed to the anterior capsule and retinaculum. The preparation of the anterior capsule and retinaculum involved the use of a rasp to promote a tissue bed that was conducive to healing. The outside-in technique using polydioxanone (PDS-II, Ethicon Inc., Raritan, NJ, USA) vertical mattress sutures was employed for all the knees. Notably, no adjunctive methodologies such as platelet-rich plasma or clot introduction were employed. Following fixation, all the repairs underwent probing and were deemed stable.

### 2.4. Postoperative Care and Rehabilitation

Following surgery, the patients were instructed to refrain from bending the knee beyond 45°. However, they were also encouraged to engage in isometric exercises targeting the quadriceps and hamstring muscles. During such exercise, patients were permitted to bear weight as tolerable. After the first month, patients were allowed to increase knee flexion progressively without specific limitations but were also advised to avoid squatting for a further 6 months. Normal walking was permitted 1 month following surgery, whereas stair climbing was permitted at 2 months post-surgery. Patients could resume their participation in sports 6 months after the surgical procedure.

### 2.5. Statistical Analysis

The repeated measures of the Lysholm Knee Scoring Scale scores were compared between two time points, namely pre-surgery and the final follow-up, by using the generalized estimating equation method. All analyses were performed using SPSS Statistics (version 28.0.1.0; IBM Corporation, Armonk, NY, USA).

## 3. Results

Between January 1993 and February 2023, 22 patients (22 knees) received a diagnosis of a hypermobile anterior horn of the lateral meniscus. After the exclusion of one knee with a discoid morphology, one knee with a history of meniscus surgery, one knee that did not undergo meniscus stabilization, one knee diagnosed as a horizontal tear of the lateral meniscus, and one knee diagnosed as having gout, 17 patients (17 knees) were consecutively included in this study. The mean follow-up period was 18.2 ± 7.6 months (range, 6–24 months). Among the analyzed patients, three (17.6%) were male, and the mean age of all the patients was 45.9 ± 18.4 years (range, 13–71 years). Two (11.8%) of the patients had a history of falls from a standing height.

Prior to surgery, all the patients exhibited anterior lateral knee pain, and 15 (88.2%) patients exhibited tenderness in the lateral joint lines as primary symptoms. Additionally, four (23.5%) patients reported experiencing a locking sensation, one (5.9%) patient exhibited hyperextension of the knee, and one (5.9%) patient experienced a loss of full extension. The median duration of symptoms was 24 months, with a range of 3 months to 6 years (Table 1).

**Figure 1:**
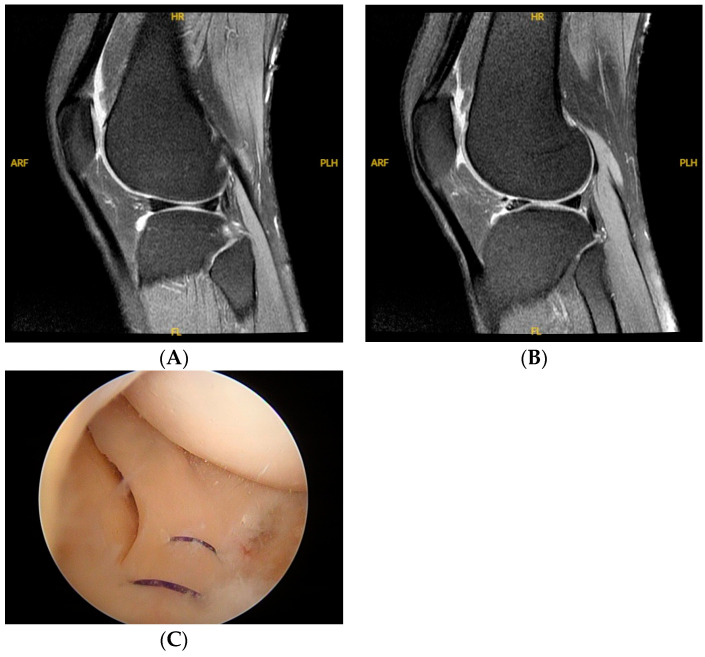
Preoperative knee magnetic resonance images of a 59-year-old female patient, and the arthroscopic image following meniscal stabilization. (**A**). The image shows the central region of the knee, which appears normal. The meniscus structure is intact, with uniform signal intensity and no abnormal high-signal areas. (**B**). The lateral side of the knee, where a hyperintense lesion is observed anterior to the meniscus. Additionally, there is fluid accumulation at the anterior horn, which may indicate synovitis or another inflammatory reaction. (**C**). Following meniscal stabilization.

Table 2 outlines the imaging findings. No abnormal findings were detected on the knee plain films. Regarding MRI, four knees exhibited completely normal results. A lateral anterior horn meniscal tear was suspected in three knees. One knee exhibited evidence of a parameniscal tear. Deformation of the anterior horn was observed in four knees. Effusion was observed in six knees, with fluid accumulation noted at the anterior horn in seven knees. A bone cyst was identified in the lateral tibial condyle of one knee as well as in the lateral femoral condyle of another knee. During arthroscopy, the anterior horn of the lateral meniscus was observed to be readily displaced beyond the lateral condyle by using a probe in all the knees. A parameniscal tear of the anterior horn of the lateral meniscus was observed in all the knees. No anterior horn meniscal tear was identified through direct visualization or probing. Additional arthroscopic findings included chondromalacia of the lateral tibial condyle in four knees, a chondral defect of the medial femoral condyle in three knees, a spur in the intercondylar notch observed in one knee, lateral tilting with chondromalacia patella observed in three knees, and chondrosis of the lateral patella with lateral tightness in one knee. Figure 1 shows the preoperative knee MRI of a 59-year-old female patient and the images under arthroscopy following meniscal stabilization.

Table 3 presents details regarding the surgical procedures. In addition to the outside-in repair, supplementary procedures were performed for lesions identified during arthroscopy. These included partial synovectomy (*n* = 16, 94.1%), microfracture (*n* = 2, 11.8%), lateral release of the patella (*n* = 1, 5.9%), excision of the medial plica (*n* = 1, 5.9%), and notchplasty with an excision of the spur over the lateral femoral condyle (*n* = 1, 5.9%).

Following meniscal stabilization, the hypermobility of the anterior horn of the lateral meniscus was resolved in all the knees. The Lysholm Knee Scoring Scale score exhibited a significant improvement from 65.8 ± 12.7 before surgery to 91.1 ± 9.6 at the final follow-up (*p* < 0.001). All the knees achieved a full range of motion at the final follow-up, and none of the patients experienced any complication or required reoperation.

## 4. Discussion

To the best of our knowledge, this study is the largest case-series to date focusing on the symptomatic hypermobility of the anterior horn of the lateral meniscus. The importance of this study lies in its comprehensive presentation of symptom characteristics and imaging findings, coupled with its thorough examination of clinical outcomes following surgery. Our findings suggest that arthroscopic outside-in meniscus stabilization demonstrates favorable outcomes for patients with a hypermobile anterior horn of the lateral meniscus.

Hypermobility of the lateral meniscus is more commonly observed in younger patients [1,2,3,4,5,6,7,11]. This association may be attributed to the presence of a discoid lateral meniscus, with one study revealing that 28.1% of the cases of discoid lateral meniscus exhibited peripheral rim instability [12]. Among our 17 cases, none involved a discoid meniscus and, notably, 10 patients were aged more than 50 years. Therefore, considering that a diagnosis of lateral meniscus hypermobility is crucial in older patients experiencing anterior lateral knee pain, particularly those with osteoarthritis, further investigations should be conducted to identify potential causes of knee pain and thus facilitate the selection of the most suitable treatment.

The MRI results revealed three cases exhibiting an anterior horn tear of the lateral meniscus and one case exhibiting a parameniscal tear. However, an arthroscopic examination revealed that all the analyzed cases presented with a parameniscal tear, with no case presenting with an anterior horn tear of the lateral meniscus. A previous study indicated that although an anterior horn tear of the lateral meniscus may be observable through an MRI, it may not be consistently detected under arthroscopic examination [13]. Accordingly, using a probe to gently manipulate the anterior horn of the lateral meniscus could potentially reveal a hypermobile anterior horn and thus indicate the necessity for appropriate intervention.

Often overlooked or misinterpreted image findings related to the hypermobility of the anterior horn of the lateral meniscus might dissuade surgeons from recommending surgical intervention. However, excessive mobility of the lateral meniscus could limit the effectiveness of conservative treatments and potentially elevate the risk of chondral injury. The present research highlighted several typical findings as follows: (1) Symptoms including lateral knee pain and tenderness over the anterior horn of the lateral meniscus; (2) normal appearance on plain knee films; and (3) MRI revealing hypodense lesions anterior to the meniscus, fluid accumulation, degenerative changes, or even anterior horn deformity.

During knee arthroscopy, manipulation of the posterior part of the lateral meniscus with a probe is intuitive and straightforward. However, dealing with the anterior part poses challenges owing to the presence of synovium, which can obscure the view. Therefore, in cases with no meniscal tear, surgeons may not be inclined to use a probe to evaluate the instability of the anterior part, potentially resulting in a missed diagnosis of excessive mobility in the anterior horn of the lateral meniscus. Furthermore, the scope is typically positioned in the anterior lateral position, limiting the field of view and making identification of the anterior horn challenging. In summary, in cases where a lesion is suspected, we recommend that surgeons reposition the scope to the anteromedial portal to obtain a clearer view of the anterior horn of the lateral meniscus.

Studies have indicated that arthroscopic meniscus fixation yields benefits for a hypermobile posterior horn of the lateral meniscus [4,7]. A one-case study supported the benefits of arthroscopic meniscus fixation for a hypermobile anterior horn of the lateral meniscus [9]. In our study of 17 cases, following meniscus stabilization, significant improvements related to both pain and function were observed; these outcomes were consistent with the findings of the aforementioned case report. In this study, the Lysholm Knee Scoring Scale was used as the primary assessment tool. This 100-point scale includes several functional parameters such as limping, pain, stability, locking, swelling, stair climbing, and squatting, making it suitable for assessing knee function after meniscus surgery. However, because the Lysholm Knee Scoring Scale does not assess the intensity of physical activities, additional assessment tools should be used for athletes.

This study had several limitations that should be acknowledged. First, no comparison of the clinical outcomes between the patients undergoing stabilization for a hypermobile anterior horn of the lateral meniscus and those not receiving stabilization was conducted; consequently, we could not assess the effectiveness of this treatment. Second, because none of the participants in this study were athletes, whether this treatment would be beneficial for enabling patients to resume engagement in high-intensity sports remains uncertain. Furthermore, a longer follow-up period is necessary to evaluate the long-term outcomes. In summary, although the results of this study may be applicable to patients with hypermobility of the anterior horn of the lateral meniscus, because of the mentioned limitations, caution is advised when interpreting these results. Accordingly, additional studies with comparative designs and larger sample sizes are warranted to validate the present findings.

## 5. Conclusions

Hypermobility in the anterior horn of the lateral meniscus is a condition that is often overlooked but can be effectively treated with meniscal stabilization. There is no specific sign or test that can be used to detect a hypermobile anterior horn of the lateral meniscus. Diagnosis must rely on arthroscopy. When a patient has lateral knee pain with a walking disability and conservative treatment is ineffective, with only vague lesions found in the anterior and lateral meniscus on MRI, a comprehensive arthroscopic assessment is crucial for accurate diagnosis of this specific pathology.

## Figures and Tables

**Table 1:** Patient Characteristics.

				Pre-Operative Symptom								
Case	Age (years)	Gender	History of Trauma	Anterior-Lateral Knee Pain	Lateral Joint Line Tenderness	Patellar Compression Pain	Locking Sensation	Effusion	Medial Joint Line Tenderness	Hyperextension Knee	Loss of Full Extension	Symptom Duration (months)
1	65	Female	Fall	+	−	−	−	−	−	−	−	7
2	59	Female	−	+	+	−	+	−	−	−	−	24
3	36	Male	−	+	+	+	+	−	−	−	−	6
4	22	Female	−	+	+	−	−	−	−	−	−	3
5	60	Female	−	+	+	−	−	−	−	−	−	6
6	57	Male	−	+	+	−	−	−	−	−	−	12
7	71	Female	−	+	+	+	+	−	−	−	+	72
8	13	Female	−	+	+	−	−	+	−	+	−	60
9	63	Female	−	+	+	+	−	+	+	−	−	24
10	56	Female	−	+	+	−	−	−	−	−	−	36
11	50	Female	−	+	+	−	+	−	−	−	−	4
12	50	Female	−	+	+	+	−	−	−	−	−	60
13	56	Female	−	+	+	−	−	−	+	−	−	60
14	18	Female	−	+	+	+	−	−	−	−	−	6
15	40	Female	−	+	+	−	−	+	−	−	−	36
16	17	Male	Fall	+	+	−	−	−	−	−	−	24
17	47	Female	−	+	−	−	−	+	−	−	−	24
Total	45.9 ± 18.4	Male: 3 (17.6%)	2 (11.8%)	17 (100%)	15 (88.2%)	5 (29.4%)	4 (23.5%)	4 (23.5%)	2 (11.8%)	1 (5.9%)	1 (5.9%)	Median: 24 (3–72)

**Table 2:** Imaging Findings.

	No. of Knees
MRI findings	
Suspected lateral anterior horn meniscus tear	3 (17.6%)
Parameniscus tear	1 (5.9%)
Deformed anterior horn	4 (23.5%)
Effusion	6 (35.3%)
Fluid accumulation noted at the anterior horn	7 (41.2%)
Bone cyst in lateral tibial condyle	1 (5.9%)
Bone cyst in lateral femoral condyle	1 (5.9%)
Arthroscopic findings	
Unstable anterior horn	17 (100%)
Parameniscus tear	17 (100%)
Chonondromalcia of lateral tibial condyle	4 (23.5%)
Chondral defect of medial femoral condyle	3 (17.6%)
Spur in intercondylar notch	1 (5.9%)
Lateral tilting with chondromalacia patella	3 (17.6%)
Chondrosis of lateral patella with lateral tightness	1 (5.9%)

**Table 3:** Surgical Procedures.

	No. of Knees
Outside-in repair	17 (100%)
2 vertical mattress sutures	6 (35.3%)
3 vertical mattress sutures	5 (29.4%)
4 vertical mattress sutures	6 (35.3%)
Additional procedures	
Partial synovectomy	16 (94.1%)
Microfracture	2 (11.8%)
Lateral release of patella	1 (5.9%)
Excision of medial plica	1 (5.9%)
Notchplasty with excision of the spur over lateral femoral condyle	1 (5.9%)

## Data Availability

The datasets generated during and analyzed during the current study are available from the corresponding author upon reasonable request.
